# Mortality burden from seasonal influenza and 2009 H1N1 pandemic influenza in Beijing, China, 2007‐2013

**DOI:** 10.1111/irv.12515

**Published:** 2017-12-02

**Authors:** Shuangsheng Wu, Zaihua Wei, Carolyn M. Greene, Peng Yang, Jianting Su, Ying Song, Angela D. Iuliano, Quanyi Wang

**Affiliations:** ^1^ Beijing Center for Disease Prevention and Control Beijing China; ^2^ Beijing Research Center for Preventive Medicine Beijing China; ^3^ United States Centers for Disease Control and Prevention Atlanta Georgia

**Keywords:** Beijing, China, H1N1 pandemic, influenza, model, mortality

## Abstract

**Background:**

Data about influenza mortality burden in northern China are limited. This study estimated mortality burden in Beijing associated with seasonal influenza from 2007 to 2013 and the 2009 H1N1 pandemic.

**Methods:**

We estimated influenza‐associated excess mortality by fitting a negative binomial model using weekly mortality data as the outcome of interest with the percent of influenza‐positive samples by type/subtype as predictor variables.

**Results:**

From 2007 to 2013, an average of 2375 (CI 1002‐8688) deaths was attributed to influenza per season, accounting for 3% of all deaths. Overall, 81% of the deaths attributed to influenza occurred in adults aged ≥65 years, and the influenza‐associated mortality rate in this age group was higher than the rate among those aged <65 years (113.6 [CI 49.5‐397.4] versus 4.4 [CI 1.7‐18.6] per 100 000, *P* < .05). The mortality rate associated with the 2009 H1N1 pandemic in 2009/2010 was comparable to that of seasonal influenza during the seasonal years (19.9 [CI 10.4‐33.1] vs 17.2 [CI 7.2‐67.5] per 100 000). People aged <65 years represented a greater proportion of all deaths during the influenza A(H1N1)pdm09 pandemic period than during the seasonal epidemics (27.0% vs 17.7%, *P* < .05).

**Conclusions:**

Influenza is an important contributor to mortality in Beijing, especially among those aged ≥65 years. These results support current policies to give priority to older adults for seasonal influenza vaccination and help to define the populations at highest risk for death that could be targeted for pandemic influenza vaccination.

## 1 INTRODUCTION

Influenza is a major cause of global morbidity and mortality each year, especially among older adults and those with chronic diseases.^1^ Although several statistical models have estimated the mortality burden of influenza,^2^ few have focused on mainland China,^3^, ^4^, ^5^ and information on mortality burden in northern China is especially limited. Considering the diverse seasonality patterns,^6^ income levels and healthcare access across China, mortality burden may vary by region.

Beijing, the capital of China, is located in northern China. With a resident population of nearly 20 million in 2010, it is one of the most populous cities in the world. During the 2009 H1N1 pandemic, only 10 844 laboratory‐confirmed cases of influenza A(H1N1)pdm09 and 69 deaths were reported in Beijing.^7^ These numbers solely represent laboratory‐confirmed influenza deaths and not decedents who might have died because of pandemic influenza but were never tested.^3^ A mathematical modeling study estimated that the likely number of influenza A (H1N1)pdm09 infections in Beijing was 1.8 million during the pandemic period.^8^ To date, no study has estimated the likely number of influenza A(H1N1)pdm09‐associated deaths in Beijing.

Since 2007, the Beijing Municipal Government has provided seasonal influenza vaccination to priority populations, including adults ≥60 years of age and primary and middle school students, free of charge between September and November each year prior to the start of typical influenza virus circulation. Beijing also conducted influenza A(H1N1)pdm09 vaccination campaigns during the pandemic period.^9^ These programs were implemented in the absence of local data on influenza‐associated mortality burden because there was concern that this novel virus could have substantial burden on the population's health. Influenza mortality burden data allow programs to determine the potential impact of influenza vaccination and inform investments in prevention strategies. In this study, we modeled mortality data obtained from a representative mortality register system, combined with weekly influenza virus surveillance data, to provide estimates of the mortality impact associated with seasonal influenza and the influenza A(H1N1)pdm09 virus during the pandemic period by specific death categories, age groups, and influenza virus type and subtype in Beijing from 2007 to 2013.

## 2 METHODS

### 2.1 Population data

We obtained annual population data from the National Population Census in Beijing, China.^10^ In 2010, the total population of Beijing was nearly 20 million, of which 12‐13 million were registered residents of Beijing. The remaining 7‐8 million persons were classified as “migrants” who are not registered residents of Beijing. In this study, we used population data of registered populations only to calculate the annual mortality rates.

### 2.2 Mortality data

Local regulation requires that all deaths in Beijing are registered in the Mortality Register and Surveillance System, managed by Beijing Center for Disease Control and Prevention (BJCDC). We were unable to obtain reliable mortality data among migrants, as both Chinese culture and high medical expenses compel a large proportion of migrants to return to their home provinces when they are severely ill, and therefore, the majority of migrants do not die in Beijing and are not registered in the BJCDC Mortality Register and Surveillance system. In this study, we obtained weekly electronic mortality data for the registered Beijing population from 2007 to 2013, covering the influenza seasons of 2007/2008 to 2012/2013. We defined an influenza season as the period ranging from the 27th week in 1 year to the 26th week in the next year, based on influenza surveillance data.^11^ The registration system categorized weekly numbers of deaths into two underlying causes of death according to the International Classification of Disease, Tenth Revision [ICD‐10]: respiratory (ICD‐10 codes J00‐99) and circulatory diseases (ICD‐10 codes I00‐99) (respiratory and circulatory: R&C), and all‐cause deaths (including both respiratory and circulatory deaths which have been previously been associated with influenza‐associated deaths).^2^, ^3^, ^4^, ^5^, ^6^ Two age groups were considered: <65 years and ≥65 years.

### 2.3 Influenza virology data

We obtained influenza virology data from the Beijing influenza surveillance system from seasons 2007/2008 to 2012/2013. The influenza surveillance system, designed and managed by BJCDC, included 14 sentinel hospitals in 2007, 17 hospitals in 2008, 20 hospitals in 2011, and 23 hospitals in 2012. In this system, clinicians of surveillance clinics in internal medicine, the emergency department, the fever clinic, and the pediatric clinic were required to diagnose all influenza‐like illness (ILI) cases (patients presenting with fever ≥38°C and cough or sore throat) and to record the weekly numbers of ILI outpatient visits throughout the year. Trained clinicians collected pharyngeal swab specimens from a convenience sample of 10‐20 ILI case‐patients per sentinel hospital per week. Specimens were sent to local CDC laboratories to test for influenza by cell culture. A more detailed description of the surveillance was published in a previous study.^12^

### 2.4 Statistical model

A negative binomial model was applied to estimate influenza‐associated excess mortality, using age‐specific weekly mortality data as the outcome and proportions of specimens positive for influenza by type/subtype (not age‐specific) as the predictor variables.^3^, ^4^, ^5^ Models included virology surveillance time series data, as well as time terms to account for the weeks in the model and harmonic terms to account for the cyclical pattern in deaths. Because of the lack of respiratory syncytial virus virology data in Beijing, we did not include this pathogen in the model.

Negative Binomial models were fit separately for the two outcomes (all‐cause and respiratory and circulatory deaths) and age groups (aged <65 years and ≥65 years) using a log link function. The final models were selected by evaluating the AIC/BIC values in combination with the statistical significance of the viral surveillance terms and the harmonic and time terms (for baseline estimation) for each of the models examined (Table S1). Further, we decided to choose the same model for each age group and outcome based on the evaluation of the separate models. We chose to use the same model for each age group for ease of interpretation and better comparison of estimates and methods. The final model selected for both all‐cause and respiratory and circulatory deaths was as follows:

$$\begin{array}{cc} Y_{t}\;=\; & \alpha \;exp\;\{\beta_{0}\;+\;\beta_{1}\left[t\right]\;+\;\beta_{2}\left[t^{2}\right]\;+\;\beta_{3}\left[\sin\left[\left(2\pi t\right)/\left(365.25/7\right)\right]\right]\\ & \;+\;\beta_{4}\left[\cos\left[\left(2\pi t\right)/\left(365.25/7\right)\right]\right]\;+\;\beta_{5}\left[A{\left(\text{H1N1}\right)}_{t}\right]\\ & \;+\;\beta_{6}\left[A{\left(\text{H3N2}\right)}_{t}\right]\;+\;\beta_{7}\left[B_{t}\right]\;+\;\beta_{8}\left[A\left(\text{H1N1}\right)pdm_{t}\right]\;+\;e_{t}\} \end{array}$$

Y_t_ is the weekly variable for the time series from 2007/2008 to 2012/2013 (1‐312) and represents age‐specific weekly number of deaths, α is the offset term equal to the log of the age‐specific population size, β_0_ represents the intercept, β_1_ and β_2_ account for the linear and quadratic time trends, β_3_ and β_4_ account for cyclical secular trends, and β_5_ through β_8_ represent coefficients associated with the weekly proportion of specimens testing positive for each influenza subtype. Further explanation of our model selection is available in Table S1.

The number of deaths attributed to influenza was calculated as the difference between the predicted influenza‐associated deaths from the full model and the predicted baseline deaths (from the model when the co‐variables for influenza subtype were set to zero). Confidence intervals were generated for excess mortality estimates based on the confidence intervals of the model predictions. Age‐specific excess mortality rates were calculated from the estimated number of deaths attributed to influenza divided by population from the National Population Census in Beijing, China.^10^ All the statistical analyses were carried out using SAS University Edition (SAS Institute Inc., Cary, NC, USA). A *P* value < .05 was considered to be statistically significant.

### 2.5 Ethics approval

Study approval was obtained from the Institutional Review Board and Human Research Ethics Committee of BJCDC.

## 3 RESULTS

### 3.1 Mortality data reported from the Mortality Register and Surveillance System and influenza virus activity, from season 2007/2008 to 2012/2013

For the influenza seasons 2007/2008 to 2012/2013, the annual all‐cause mortality rate ranged from 581.7 to 602.6 per 100 000 persons for all ages, and 3238.9 to 3394.3 per 100 000 for adults aged ≥65 years. Adults aged ≥65 years accounted for 76% of all‐cause deaths among all ages. R&C causes represented 58% of all deaths. The annual R&C mortality rate ranged from 335.8 to 346.9 per 100 000 persons for all ages and 2050.0 to 2221.0 per 100 000 for adults aged ≥65 years. Adults aged ≥65 years accounted for 84% of R&C deaths among all ages (Table 1).

**Table 1:** Mortality rates (per 100 000 persons) reported from the Mortality Register and Surveillance System by age, Beijing 2007/2008 to 2012/2013 influenza seasons

	All ages			Adults aged ≥65 y (% of deaths among all age)		
	Population	All‐cause rate	R&C rate (% of all‐cause deaths	Population	All‐cause	R&C
2007/2008	12 047 617	581.7	343.4 (59)	1 560 836	3394.3 (75.6)	2221 (83.8)
2008/2009	12 208 706	575	335.8 (58.4)	1 614 871	3292.6 (75.7)	2113.7 (83.3)
2009/2010	12 375 798	602.6	354.2 (58.8)	1 664 285	3388.4 (75.6)	2196.3 (83.4)
2010/2011	12 518 114	593.5	346.9 (58.4)	1 707 318	3329.7 (76.5)	2143.8 (84.3)
2011/2012	12 678 603	595.3	340.8 (57.2)	1 764 450	3274.3 (76.5)	2064.0 (84.3)
2012/2013	12 876 915	601.9	346.2 (57.5)	1 840 459	3238.9 (76.9)	2050.0 (84.6)

R&C, Respiratory and circulatory diseases.

**Figure 1:**
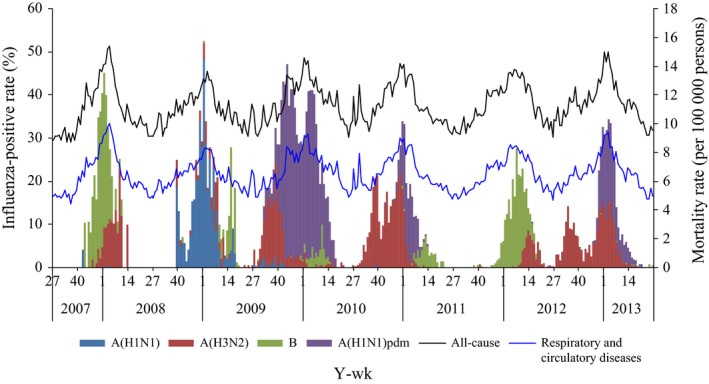
Weekly mortality rates reported from the Mortality Register and Surveillance System and proportions of specimens positive for influenza, Beijing, 2007/2008 to 2012/2013

During the 6‐year study period, a total of 60 795 specimens were tested for influenza virus. An average of 15.4% specimens tested positive for influenza for the entire time series. Influenza A(H1N1), A(H1N1)pdm09, and A(H3N2) were each the predominant subtype in one season (2008/2009, 2009/2010, and 2010/2011, respectively), while influenza B predominated in 2 seasons (2007/2008 and 2011/2012), and A(H1N1)pdm09 and A(H3N2) co‐circulated in the 2012/2013 season (Figure 1). Influenza virus activity and mortality both peaked in the winter months of December‐February during the non‐pandemic period and from November of 2009 to February of 2010 during the pandemic period (Figure 1).

### 3.2 Estimates of influenza‐associated deaths, from season 2007/2008 to 2012/2013

**Figure 2:**
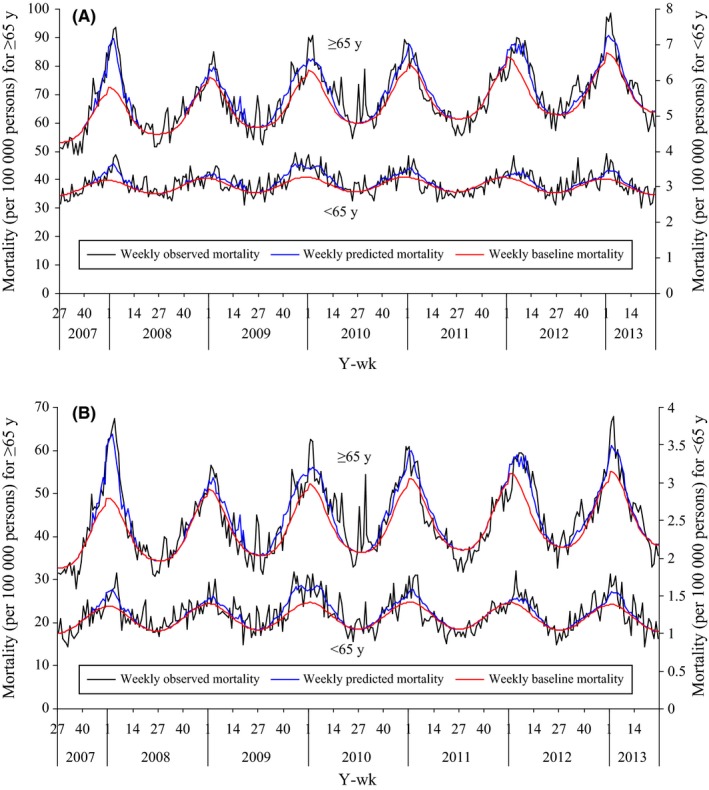
Weekly observed, predicted, and baseline mortality by negative binomial regression model, Beijing, 2007/2008 to 2012/2013. Panel (A) all cause; panel (B) respiratory and circulatory diseases

Figure 2 presents the weekly time series of observed, predicted, and baseline mortality using a negative binomial regression model. The predicted values fit well with the observed data.

For all ages, there was year‐to‐year variability in the annual number of influenza‐associated all‐cause deaths, and the mean number of deaths from 2007/2008 to 2012/2013 was 2375(95% confidence interval [CI] 1002‐8688), with a rate of 19.1 (CI 8.1‐69.9) deaths per 100 000 persons (Table 2). Most (81%) influenza‐associated all‐cause deaths occurred among adults aged ≥65 years, and the rate in this age group was higher than the rate among those aged <65 years (113.6 [CI 49.5‐397.4] versus 4.4 [CI 1.7‐18.6] per 100 000, *P* < .05). There were, on average, 1873 (CI 818‐6684) influenza‐associated R&C deaths per year, with a rate of 15.1 (CI 6.6‐53.8) per 100 000. Influenza‐associated R&C mortality varied by season (ranged from 8.9 [CI 1.7‐48.1] to 23.2 [CI 10.5‐64] per 100 000) and 87% of deaths occurred in those aged ≥65 years.

**Table 2:** Influenza‐associated excess deaths and influenza‐associated death rates (per 100 000 persons) by age, Beijing, 2007/2008 to 2012/2013

Season	Influenza‐associated excess deaths			Influenza‐associated death rates		
	All ages^a^	Age≥65 y		All ages^a^	Age ≥65 y	Age <65 y
	No.(CI)	No. (CI)	% of all ages^b^	rates(CI)	rates(CI)	rates(CI)
All‐cause						
2007/2008	3039 (1788‐9984)	2562 (1579‐7803)	84	25.2 (14.8‐82.9)	164.1 (101.2‐499.9)	4.5 (2‐20.8)
2008/2009	1532 (317‐7787)	1292 (286‐6000)	84	12.5 (2.6‐63.8)	80 (17.7‐371.5)	2.3 (0.3‐16.9)
2009/2010	3576 (1544‐10126)	2610 (1039‐7549)	73	28.9 (12.5‐81.8)	156.8 (62.4‐453.6)	9 (4.7‐24.1)
2010/2011	2101 (707‐7740)	1670 (591‐5950)	79	16.8 (5.6‐61.8)	97.8 (34.6‐348.5)	4 (1.1‐16.6)
2011/2012	2225 (1304‐8093)	1893 (1164‐6371)	85	17.5 (10.3‐63.8)	107.3 (66‐361.1)	3 (1.3‐15.8)
2012/2013	1777 (350‐8396)	1391 (280‐6432)	78	13.8 (2.7‐65.2)	75.6 (15.2‐349.5)	3.5 (0.6‐17.8)
Mean (all years)	2375 (1002‐8688)	2375 (1002‐8688)	81	19.1 (8.1‐69.9)	113.6 (49.5‐397.4)	4.4 (1.7‐18.6)
Mean (seasonal years^c^)	2135 (893‐8400)	2135 (893‐8400)	82	17.2 (7.2‐67.5)	105 (46.9‐386.1)	3.5 (0.9‐14.6)
R&C						
2007/2008	2194 (1344‐6467)	2194 (1344‐6467)	91	20.1 (11.6‐64.7)	140.6 (86.1‐414.3)	2.1 (0.6‐12.7)
2008/2009	957 (201‐4747)	957 (201‐4747)	88	8.9 (1.7‐48.1)	59.3 (12.4‐294)	1.2 (0.1‐10.7)
2009/2010	2284 (995‐6271)	2284 (995‐6271)	79	23.2 (10.5‐64)	137.2 (59.8‐376.8)	5.5 (2.9‐15.4)
2010/2011	1489 (613‐4907)	1489 (613‐4907)	86	13.8 (5.3‐48.1)	87.2 (35.9‐287.4)	2.2 (0.5‐10.3)
2011/2012	1524 (931‐5061)	1524 (931‐5061)	92	13.1 (7.6‐47.9)	86.4 (52.8‐286.8)	1.3 (0.3‐9.3)
2012/2013	1248 (329‐5215)	1248 (329‐5215)	85	11.4 (2.8‐49.9)	67.8 (17.9‐283.4)	2 (0.3‐10.9)
Mean (all years)	1873 (818‐6684)	1616 (736‐5445)	87	15.1 (6.6‐53.8)	96.4 (44.1‐323.8)	2.4 (0.8‐11.5)
Mean (seasonal years^c^)	1673 (721‐6437)	1482 (684‐5279)	88	13.5 (5.8‐51.7)	88.2 (41‐313.2)	1.8 (0.3‐9)

CI, 95% confidence interval; R&C, Respiratory and circulatory diseases.

a The sum of the estimates of influenza‐associated deaths for persons aged ≥65 y and persons aged<65 y.

b % of all ages = Influenza‐associated excess deaths for persons aged ≥65 y ÷ Influenza‐associated excess deaths for all ages.

c seasonal years: all years excluding 2009/2010.

Table 3 lists influenza death rates by virus type/subtype from season 2007/2008 to 2012/2013. For all ages, the mortality rate associated with influenza B was 7.7 (CI 4.6‐20.3) per 100 000 persons per season for all‐cause mortality, followed by A(H1N1)pdm09 (7.1 [CI 4.1‐19.3] per 100 000 for 2009/2010‐2012/2013) and A(H3N2) (5.6 [CI 1.4‐18.3] per 100 000). Influenza B caused the highest mortality burden (20.5 [CI 14.2‐34.9] per 100 000) when influenza B virus predominated in 2007/2008. A similar pattern was observed among both those aged <65 years and those aged ≥65 years, and also within all respiratory and circulatory deaths (Table 4).

**Table 3:** Influenza‐associated death rates (per 100 000 persons) by virus subtype, Beijing, 2007/2008 to 2012/2013

Season	All	Rates (% of deaths) by virus subtypes				The predominant virus circulating
		A(H1N1)	A(H3N2)	B	A(H1N1)pdm09	
All‐cause						
2007/2008	25.2	0.1 (0.3)	4.6 (18.3)	20.5 (81.4)	0 (0)	B
2008/2009	12.5	6.4 (51)	2.2 (17.4)	4 (31.5)	0 (0)	A(H1N1)
2009/2010	28.9	0.3 (0.9)	6 (20.7)	2.7 (9.5)	19.9 (68.9)	A(H1N1)pdm
2010/2011	16.8	0 (0.1)	10.3 (61.3)	2.9 (17.6)	3.5 (21.1)	A(H3N2)
2011/2012	17.5	0 (0)	1.9 (11.1)	15.6 (88.8)	0 (0.1)	B
2012/2013	13.8	0 (0)	8.7 (63)	0.2 (1.2)	4.9 (35.8)	A(H1N1)pdm, A(H3N2)
Mean (all years)	19.1	1.1 (8.7)	5.6 (32)	7.7 (38.3)	7.1 (36.6)^a^	
Mean (seasonal years^b^)	17.2	1.3 (5.2)	5.5 (32.6)	8.6 (49.9)	2.8 (17.7)^a^	
R&C						
2007/2008	20.1	0.1 (0.3)	4.1 (20.6)	15.9 (79.1)	0 (0)	B
2008/2009	8.9	4.2 (46.9)	1.9 (21.3)	2.8 (31.9)	0 (0)	A(H1N1)
2009/2010	23.2	0.1 (0.6)	5 (21.7)	2.1 (8.8)	16 (68.9)	A(H1N1)pdm
2010/2011	13.8	0 (0.1)	8.8 (64)	2.1 (15.3)	2.8 (20.7)	A(H3N2)
2011/2012	13.1	0 (0)	1.6 (12.3)	11.5 (87.7)	0 (0)	B
2012/2013	11.4	0 (0)	7.4 (64.9)	0.1 (0.9)	3.9 (34.2)	A(H1N1)pdm, A(H3N2)
Mean (all years)	15.1	0.7 (8)	4.8 (34.1)	5.7 (37.3)	5.7 (36.7)^a^	
Mean (seasonal years^b^)	13.5	0.5 (6.2)	4.7 (35.8)	6.5 (47.8)	2.3 (17.7)^a^	

Number (proportion) presented.

R&C, Respiratory and circulatory diseases.

a Estimates for seasons 2009 to 2013.

b seasonal years: all years excluding 2009/2010.

**Table 4:** Influenza‐associated death rates (per 100 000 persons) by virus subtype and by age group, Beijing, 2007/2008 to 2012/2013

Season	Age ≥65 y					Age <65 y				
	All	Rates (% of deaths) by virus subtypes				All	Rates (% of deaths) by virus subtypes			
		A(H1N1)	A(H3N2)	B	A(H1N1)pdm09		A(H1N1)	A(H3N2)	B	A(H1N1)pdm09
All‐cause										
2007/2008	164.1	0.6 (0.4)	29 (17.6)	134.6 (82)	0 (0)	4.5	0 (0.2)	1 (21.6)	3.6 (78.2)	0 (0)
2008/2009	80.0	41.4 (51.8)	13.5 (16.9)	25.1 (31.3)	0 (0)	2.3	1.1 (47.1)	0.5 (20.4)	0.7 (32.5)	0 (0)
2009/2010	156.8	1.7 (1.1)	36 (23)	17.2 (11)	101.8 (64.9)	9.0	0 (0.2)	1.3 (14.8)	0.5 (5.4)	7.2 (79.6)
2010/2011	97.8	0.1 (0.1)	61.4 (62.8)	18.2 (18.6)	18 (18.4)	4.0	0 (0)	2.2 (55.2)	0.5 (13.5)	1.2 (31.3)
2011/2012	107.3	0.1 (0.1)	11.4 (10.7)	95.7 (89.2)	0.1 (0.1)	3.0	0 (0)	0.4 (13.3)	2.6 (86.7)	0 (0)
2012/2013	75.6	0 (0)	50 (66.1)	1 (1.4)	24.6 (32.5)	3.5	0 (0)	1.8 (51.8)	0 (0.5)	1.7 (47.7)
Mean(all years)	113.6	7.3 (8.9)	33.6 (32.8)	48.6 (38.9)	36.2 (32.5)^a^	4.4	0.2 (7.9)	1.2 (29.5)	1.3 (36.1)	2.5 (39.6)^a^
Mean (seasonal years^b^)	105.0	8.4 (7.7)	33.1 (32.3)	54.9 (51.4)	14.2 (15.4)^a^	3.5	0.2 (6.1)	1.2 (34)	1.5 (42.8)	1.0 (26.3)^a^
R&C										
2007/2008	140.6	0.4 (0.3)	28.3 (20.1)	111.8 (79.5)	0 (0)	2.1	0 (0.4)	0.5 (24.6)	1.6 (75)	0 (0)
2008/2009	59.3	27.1 (45.8)	12.7 (21.4)	19.4 (32.8)	0 (0)	1.2	0.7 (55)	0.2 (20.2)	0.3 (24.8)	0 (0)
2009/2010	137.2	1 (0.7)	33 (24.1)	13.9 (10.1)	89.3 (65.1)	5.5	0 (0)	0.7 (12.5)	0.2 (3.9)	4.6 (83.6)
2010/2011	87.2	0.1 (0.1)	57.1 (65.5)	14.1 (16.2)	15.9 (18.3)	2.2	0 (0)	1.2 (54.3)	0.2 (9.8)	0.8 (35.9)
2011/2012	86.4	0 (0)	10.4 (12.1)	75.9 (87.9)	0 (0)	1.3	0 (0)	0.2 (14.9)	1.1 (85.1)	0 (0)
2012/2013	67.8	0 (0)	46.1 (67.9)	0.7 (1)	21 (31)	2.0	0 (0)	1 (48.2)	0 (0)	1.1 (51.8)
Mean (all years)	96.4	4.8 (7.8)	31.3 (35.2)	39.3 (37.9)	31.6 (32.8)^a^	2.4	0.1 (9.2)	0.6 (29.1)	0.6 (33.1)	1.6 (42.8)^a^
Mean (seasonal years^b^)	88.2	5.5 (6)	30.9 (35.8)	44.4 (49.2)	12.3 (15.5)^a^	1.8	0.1 (7.6)	0.6 (35.4)	0.6 (36)	0.6 (29.2)^a^

Number (proportion) presented.

R&C, Respiratory and circulatory diseases.

a Estimates for seasons 2009 to 2013.

b seasonal years: all years excluding 2009/2010.

### 3.3 Comparisons of mortality burden between seasonal epidemics and the influenza A(H1N1)pdm09 pandemic period

Table 5 shows comparisons of mortality burden between seasonal epidemics and the influenza A(H1N1)pdm09 pandemic period from this study and previous studies. The all‐cause mortality rate associated with the influenza A(H1N1)pdm09 was comparable to the mortality rate of seasonal influenza during the non‐pandemic seasons (19.9 [CI 10.4‐33.1] vs 17.2 [CI 7.2‐67.5] per 100 000). The mortality impact of the influenza A(H1N1)pdm09 was about double that of seasonal epidemics in non‐pandemic seasons among persons aged <65 years (7.2 [CI 4.4‐10.9] vs 3.5 [CI 0.9‐14.6] per 100 000), but almost equal among older adults (101.8 [CI 48.4‐175.9] vs 105 [CI 46.9‐386.1] per 100 000). However, the differences did not reach statistical significance (*P* > .05). Persons aged <65 years represented a greater proportion of all deaths during the influenza A(H1N1)pdm09 pandemic period than during the seasonal epidemics (27.0% vs 17.7%, *P* < .05). A similar mortality burden age shift toward persons <65 years of age was also observed for respiratory and circulatory deaths.

**Table 5:** Comparisons of influenza‐associated death rates (per 100 000 persons) by age and death category in this study with previous studies from China

	Model^a^	Study period	All ages	Age ≥65 y	Age <65 y	% of deaths in age <65 y^b^
All deaths: seasonal						
China(Beijing, this study)	NB	2007‐2013	17.2 (7.2‐67.5)	105 (46.9‐386.1)	3.5 (0.9‐14.6)	17.7
China(Northern cities)^3^	NB	2003‐2008	18 (10.9‐32.7)	150.8 (66.2‐502.5)	1.3	6.3
China(Southern cities)^3^	NB	2003‐2008	11.3 (1.4‐50.4)	75.4 (11.0‐313.6)	1.8	13.7
China^4^	NB	2004‐2010	15.1 (6.3‐48.9)	145.5 (95.8‐227.3)	3.7 (1.2‐14.8)	22.4
China(Guangzhou city)^13^	P	2004‐2006	10.6 (0.6‐17.3)	111.3 (19.4‐203.2)	‐	‐
China(Guangzhou city)^5^	NB	2010‐2012	14.7 (12.1‐17.3)	185.6 (152.5‐218.7)	2.5 (2‐3)	34.8
Hong Kong, China^14^	P	1996‐1999	16.4 (9.4‐23.3)	136.1 (83.7‐188.4)	11.8 (3.8‐20.1)	21.2
Hong Kong, China^13^	P	2004‐2006	13.4 (5.4‐20.9)	103.7 (40.2‐117.1)	‐	‐
All deaths: 2009 pandemic						
China(Beijing, this study)	NB	2007‐2013	19.9 (10.4‐33.1)	101.8 (48.4‐175.9)	7.2 (4.4‐10.9)	27.0
China^4^	NB	2004‐2010	13.8 (6.3‐28.1)	125.8 (59.3‐250.2)	3.9 (1.7‐8.6)	26.3
Hong Kong, China^15^	P	1998‐2009	1.8 (0‐3.7)	‐	‐	8.7
R&C Deaths: Seasonal						
China(Beijing, this study)	NB	2007‐2013	13.5 (5.8‐51.7)	88.2 (41‐313.2)	1.8 (0.3‐9)	11.7
China(Northern cities)^3^	NB	2003‐2008	12.4 (7.4‐22.2)	106 (46.7‐344.1)	0.6	4.3
China(Southern cities)^3^	NB	2003‐2008	8.8 (1.8‐35.1)	64.3 (14.0‐243.7)	0.6	6
China^4^	NB	2004‐2010	11.1 (5‐32.2)	117.8 (54.8‐329.3)	1.7 (0.6‐6.1)	14.5
China(Guangzhou city)^13^	P	2004‐2006	9.9 (2‐17.6)	104.1 (27.5‐177.5)	‐	‐
China(Guangzhou city)^5^	NB	2010‐2012	11.4 (9.4‐13.4)	146.9 (120.7‐173)	1.7 (1.4‐2)	18
Hong Kong, China^14^	P	1996‐1999	12.4 (7.7‐17.1)	102.4 (61.2‐142.7)	7.3 (3.1‐11.4)	17.2
Hong Kong, China^13^	P	2004‐2006	9.5 (4.5‐14.6)	78.7 (40.2‐117.1)	‐	‐
R&C deaths: 2009 pandemic						
China(Beijing, this study)	NB	2007‐2013	16 (8.6‐26.2)	89.3 (45.5‐149.3)	4.6 (2.8‐7.1)	20.6
China^4^	NB	2004‐2010	9.4 (4.6‐18.6)	94.6 (44.5‐187.1)	2 (1‐3.8)	19
Hong Kong, China^15^	P	1998‐2009	1.6 (0.4‐2.9)	‐	‐	15.7

rate (95% confidence interval) presented.

a NB denotes negative binomial model; P denotes Poisson model.

b % of deaths in persons aged<65 years was a calculation of estimated numbers of influenza.

## 4 DISCUSSION

This study estimated the mortality burden of seasonal influenza 2007/2008‐2012/2013 and influenza A(H1N1)pdm09 in Beijing based on robust vital statistics and mortality data. We estimated an average of 2375 (CI 1002‐8688) influenza‐associated all‐cause deaths per year, accounting for 3% (CI 2‐4%) of all reported deaths from the Mortality Register and Surveillance System. All‐cause mortality rates associated with influenza A(H1N1)pdm09 and seasonal influenza 2007/2008‐2012/2013 were 19.9 (CI 10.4‐33.1) and 17.2 (CI 7.2‐67.5) per 100 000 persons, respectively.

Our estimates are consistent with previous studies that have shown that the majority of influenza‐associated deaths occurred in persons aged ≥65 years (Table 5).^3^, ^4^, ^5^, ^13^, ^14^, ^15^, ^16^, ^17^, ^18^, ^19^, ^20^, ^21^, ^22^, ^23^ Notably, our study observed that influenza B caused the highest burden (20.5 [CI 14.2‐34.9] per 100 000) when influenza B virus predominated in 2007/2008. This mortality pattern is consistent with those described in studies conducted by China CDC and Guangzhou CDC,^3^, ^5^ but differs from studies conducted in other regions such as Hong Kong,^14^ Singapore,^16^ Thailand,^17^ United States,^18^, ^19^ and New Zealand,^23^ where the highest death rates were associated with influenza A(H3N2). In addition, the highest mortality burden was not observed in 2011/2012 when influenza B also predominated in Beijing. In 2007/2008, the circulating influenza B viruses originated from the B/Yamagata/16/88 lineage and did not match the vaccine strain, which originated from a different and antigenically distinct lineage of influenza B virus (B/Victoria/2/87).^24^ The mismatch of the influenza B strain included in the Northern Hemisphere vaccine with the circulating influenza B strain had a negative impact on the effectiveness of the vaccine in the targeted population.^25^ In contrast, the 2012/2013 influenza vaccine was moderately effective against influenza B.^26^ Thus, the very low vaccine effectiveness may have led to a higher influenza B infection incidence and mortality in 2007/2008.

Our findings for the all‐age mortality burden of seasonal influenza are comparable with previous studies derived from similar approaches in China (Table 5) 3, 4, 5 and other areas, such as Hong Kong,^14^ Singapore,^16^ USA,^18^ and Mexico.^20^ For adults aged ≥65 years, however, our estimate is lower than previous estimates made for China overall, 3 northern cities in China, and a southern city in China (105 [46.9‐386.1] vs 145.5 [95.8‐227.3], 150.8 [66.2‐502.5], and 185.6 [152.5‐218.7] per 100 000, respectively).^3^, ^4^, ^5^ One explanation for this lower burden of seasonal influenza mortality among older adults in Beijing may be the higher seasonal influenza vaccine coverage among older adults in Beijing compared with older adults in the rest of China. Since 2007, the Beijing Municipal Government has provided annual seasonal influenza vaccination to older adults free of charge, and the vaccine coverage rate for this population in season 2010/2011 was 43%.^9^ In most regions of China, older adults must pay for the seasonal influenza vaccine out of pocket, leading to very low vaccine coverage. One study estimated that the vaccine coverage in the Chinese population overall was only 2% in 2009.^27^ Second, as the largest proportion of influenza‐associated deaths among older adults is among those aged ≥75 years,^18^ different age distributions across regions may contribute to differences in influenza‐associated mortality burden. Beijing is a large city with a very high population aging rate that has reached the average level of developed countries.^28^ A higher proportion of adults aged ≥75 years in Beijing may contribute to a higher mortality burden among all adults aged ≥65 years. However, as Beijing is also one of the most developed regions in China, on average, its residents have higher socioeconomic status and better access to health care than residents in other Chinese regions which, in turn, may contribute to a lower mortality burden among older adults.

We estimated that the all‐cause mortality rate associated with influenza A(H1N1)pdm09 was comparable to the mortality impact of seasonal epidemics during the non‐pandemic seasons (19.9 vs 17.2 per 100 000). Although the influenza A(H1N1)pdm09 mortality rate in this study is similar to that estimated by another study in China,^4^ it is lower than the rate described in Mexico,^20^ and higher than rates estimated in the United States.^29^ The case fatality rate of pH1N1 infection during the pandemic was higher in Mexico than in Beijing (1.2% vs 0.6%),^30^ contributing to the higher influenza A(H1N1)pdm09 mortality rate described in Mexico. Many factors may have contributed to the regional difference in the mortality impact of the pandemic virus between Mexico and Beijing, including epidemic intensity, use of influenza vaccine and antiviral drugs, and healthcare access. Meanwhile, the lower rates described in the United States may have been associated with the higher vaccination coverage for influenza A(H1N1)pdm09 achieved in the United States compared with Beijing (20% vs 13%, respectively).^31^, ^32^ Finally, treatment factors may have contributed to different pandemic mortality rates in the United States and Beijing. Due to limited supply of antiviral drugs in Beijing, most patients with mild influenza illness did not take neuraminidase inhibitors within 48 hours of illness onset, increasing the likelihood of severe or fatal outcomes.^7^ In addition, although approximately 80% of patients with severe illness from influenza A(H1N1)pdm09 in Beijing were treated with neuraminidase inhibitors at some point during the course of their illness, in most cases, treatment was not initiated within 48 hours of illness onset.^7^

Consistent with studies conducted in other countries, our study found that, compared with seasonal epidemics, there was an “age shift” in age‐specific mortality burden toward persons <65 years of age during the influenza A(H1N1)pdm09 pandemic period.^4^, ^20^, ^29^, ^33^, ^34^, ^35^, ^36^ A previous meta‐analysis from 27 studies showed that the influenza A(H1N1)pdm09 cumulative incidence varied significantly by age with much higher incidence in younger people.^37^ In addition, a study comparing seasonal influenza and influenza A(H1N1)pdm09 demonstrated that younger people had 2‐4 times the risk of severe outcomes from influenza A(H1N1)pdm09 than persons of the same ages with seasonal influenza.^38^ These findings suggest that prevention and control measures during pandemics may need to target younger adults who are not considered high risk during seasonal epidemics.

This study has several limitations. First, although underreporting of deaths in Beijing is low when compared to China overall (4‐7% vs 17%),^39^, ^40^, ^41^ it is possible that underreporting still led to an underestimation of influenza mortality. Second, because Beijing does not have access to local respiratory syncytial virus (RSV) virology data, we were unable to control for RSV co‐circulation. As RSV is associated with substantial mortality,^18^ not accounting for RSV may have inflated our influenza‐related mortality burden estimate. While it is possible that our estimates might be an overestimate of influenza‐associated deaths, the inclusion of viral surveillance terms significantly reduces the chance of overestimation. Prior to including these terms, we evaluated the surveillance data to ensure that it was robust and did not show instances of bias due to few specimens being collected. We also compared our viral surveillance data to the national system which showed a similar pattern in influenza virus circulation for the northern region of China. Third, we did not have the sample size available to obtain reliable estimates within more refined age categories. There were also several factors that may contribute to deaths in Beijing that we were unable to include in our model, such as air quality, which is particularly poor in Beijing especially when compared to cities outside of China. Finally, our estimates were based on an ecological study design, and therefore, we cannot confirm a causal relationship between influenza activity and our estimates of mortality burden.

## 5 CONCLUSIONS

Our study demonstrates a substantial influenza‐related mortality burden in Beijing, China from 2007/2008 to 2012/2013, especially among older adults. Influenza B caused highest mortality when influenza B virus was the predominant circulating virus in 2007/2008. Although this study did not find any increase in all‐age influenza‐associated mortality during the influenza A(H1N1)pdm09 pandemic period, the mortality burden in 2009/2010 increased among persons aged <65 years. These findings inform local influenza prevention and control strategies. First, the results support policies that give priority to older adults for seasonal influenza vaccination as they have done since 2007, and efforts to identify strategies to increase vaccine coverage in this population at highest risk for seasonal influenza‐related mortality. Second, the increased influenza‐associated mortality rates among persons <65 years of age during the influenza A(H1N1)pdm09 pandemic period suggest that the most effective pandemic vaccination strategies may differ from those used for seasonal influenza; it is important to identify and vaccinate populations at highest risk for morbidity and mortality during each pandemic.

## CONFLICT OF INTEREST

The authors declare that they have no competing interests.

## AUTHORS’ CONTRIBUTION

WS, WZ, YP, and WQ designed the study; WS and SJ performed the data collection; WQ and WZ coordinated and supervised the data collection; WS analyzed the data; WS, YP, CG, SY, and AI participated in the interpretation of data; WS drafted the initial manuscript; WS, CG, SY, and AI revised the manuscript. All authors have read and approved the final version of the manuscript.
