# Supplementary material for: Mortality burden from seasonal influenza and 2009 H1N1 pandemic influenza in Beijing, China, 2007‐2013
Source: Influenza Other Respir Viruses. 2017 Dec 2;12(1):88–97. doi: 10.1111/irv.12515 (PMC5818349; doi:10.1111/irv.12515)
Supplement: Supplementary file 1 [file IRV-12-88-s001.doc]

**Supplementary tables**

Table S1. Beta values, p-values and AIC/BIC values for the models.

| Models | Variables in the model |  | time | square | cube | cos1 | sin1 | cos2 | sin2 | H1 | H3 | H1pdm | B | AIC | AICC | BIC | Chosen model |
| --- | --- | --- | --- | --- | --- | --- | --- | --- | --- | --- | --- | --- | --- | --- | --- | --- | --- |
| Regression model for all-cause deaths for people aged >=65 years | | | | | | | | | | | | | |  | | | |
| Model 1 | time square cube cos1 sin1 cos2 sin2 H1 H3 H1pdm B | beta values | 0.001 | 0.000 | 0.000 | -0.136 | 0.000 | 0.025 | 0.002 | 0.000 | 0.003 | 0.001 | 0.005 | 3535.2 | 3536.4 | 3583.8 |  |
|  |  | p-value | 0.021 | 0.062 | 0.171 | 0.000 | 0.997 | 0.000 | 0.711 | 0.581 | 0.000 | 0.023 | 0.000 |  |  |  |  |
| Model 2 | time square cos1 sin1 cos2 sin2 H1 H3 H1pdm B | beta values | 0.000 | 0.000 |  | -0.134 | -0.002 | 0.024 | 0.001 | 0.001 | 0.003 | 0.001 | 0.005 | 3535.1 | 3536.1 | 3580.0 |  |
|  |  | p-value | 0.003 | 0.000 |  | 0.000 | 0.785 | 0.000 | 0.892 | 0.254 | 0.000 | 0.004 | 0.000 |  |  |  |  |
| Model 3 | time square cos1 sin1 H1 H3 H1pdm B | beta values | 0.000 | 0.000 |  | -0.132 | 0.001 |  |  | 0.001 | 0.003 | 0.002 | 0.006 | 3552.4 | 3553.2 | 3589.9 | final model |
|  |  | p-value | 0.005 | 0.001 |  | 0.000 | 0.919 |  |  | 0.067 | 0.000 | 0.000 | 0.000 |  |  |  |  |
| Regression model for all-cause deaths for people aged <65 years | | | | | | | | | | | | | |  | | | |
| Model 1 | time square cube cos1 sin1 cos2 sin2 H1 H3 H1pdm B | beta values | 0.001 | 0.000 | 0.000 | -0.069 | 0.012 | 0.004 | -0.005 | 0.001 | 0.002 | 0.002 | 0.004 | 2852.5 | 2853.7 | 2901.1 |  |
|  |  | p-value | 0.059 | 0.158 | 0.284 | 0.000 | 0.051 | 0.420 | 0.424 | 0.575 | 0.008 | 0.000 | 0.000 |  |  |  |  |
| Model 2 | time square cos1 sin1 cos2 sin2 H1 H3 H1pdm B | beta values | 0.000 | 0.000 |  | -0.067 | 0.011 | 0.004 | -0.006 | 0.001 | 0.002 | 0.003 | 0.004 | 2851.6 | 2852.7 | 2896.6 |  |
|  |  | p-value | 0.010 | 0.015 |  | 0.000 | 0.075 | 0.453 | 0.314 | 0.296 | 0.004 | 0.000 | 0.000 |  |  |  |  |
| Model 3 | time square cos1 sin1 H1 H3 H1pdm B | beta values | 0.000 | 0.000 |  | -0.068 | 0.011 |  |  | 0.001 | 0.002 | 0.003 | 0.003 | 2849.2 | 2850.0 | 2886.7 | final model |
|  |  | p-value | 0.009 | 0.014 |  | 0.000 | 0.073 |  |  | 0.311 | 0.007 | 0.000 | 0.000 |  |  |  |  |
| Regression model for R&C deaths for people aged >=65 years | | | | | | | | | | | | | |  | | | |
| Model 1 | time square cube cos1 sin1 cos2 sin2 H1 H3 H1pdm B | beta values | 0.001 | 0.000 | 0.000 | -0.182 | -0.014 | 0.028 | 0.002 | 0.000 | 0.004 | 0.002 | 0.007 | 3390.0 | 3391.2 | 3438.6 |  |
|  |  | p-value | 0.049 | 0.069 | 0.159 | 0.000 | 0.044 | 0.000 | 0.796 | 0.805 | 0.000 | 0.010 | 0.000 |  |  |  |  |
| Model 2 | time square cos1 sin1 cos2 sin2 H1 H3 H1pdm B | beta values | 0.000 | 0.000 |  | -0.179 | -0.016 | 0.028 | 0.000 | 0.001 | 0.004 | 0.002 | 0.007 | 3390.0 | 3391.0 | 3434.9 |  |
|  |  | p-value | 0.061 | 0.003 |  | 0.000 | 0.020 | 0.000 | 0.985 | 0.405 | 0.000 | 0.001 | 0.000 |  |  |  |  |
| Model 3 | time square cos1 sin1 H1 H3 H1pdm B | beta values | 0.000 | 0.000 |  | -0.177 | -0.014 |  |  | 0.001 | 0.004 | 0.002 | 0.007 | 3403.8 | 3404.5 | 3441.2 | final model |
|  |  | p-value | 0.073 | 0.006 |  | 0.000 | 0.054 |  |  | 0.145 | 0.000 | 0.000 | 0.000 |  |  |  |  |
| Regression model for R&C deaths for people aged <65 years | | | | | | | | | | | | | |  | | | |
| Model 1 | time square cube cos1 sin1 cos2 sin2 H1 H3 H1pdm B | beta values | 0.003 | 0.000 | 0.000 | -0.151 | 0.005 | 0.010 | -0.005 | 0.000 | 0.002 | 0.003 | 0.004 | 2557.1 | 2558.4 | 2605.8 |  |
|  |  | p-value | 0.002 | 0.002 | 0.006 | 0.000 | 0.594 | 0.260 | 0.584 | 0.870 | 0.051 | 0.000 | 0.001 |  |  |  |  |
| Model 2 | time square cos1 sin1 cos2 sin2 H1 H3 H1pdm B | beta values | 0.000 | 0.000 |  | -0.143 | 0.000 | 0.009 | -0.010 | 0.001 | 0.003 | 0.004 | 0.004 | 2562.7 | 2563.8 | 2607.6 |  |
|  |  | p-value | 0.133 | 0.045 |  | 0.000 | 0.993 | 0.334 | 0.293 | 0.333 | 0.014 | 0.000 | 0.002 |  |  |  |  |
| Model 3 | time square cos1 sin1 H1 H3 H1pdm B | beta values | 0.000 | 0.000 |  | -0.145 | 0.000 |  |  | 0.001 | 0.003 | 0.004 | 0.004 | 2560.8 | 2561.5 | 2598.2 | final model |
|  |  | p-value | 0.126 | 0.044 |  | 0.000 | 0.965 |  |  | 0.339 | 0.026 | 0.000 | 0.003 |  |  |  |  |

NOTE: time=t, square=t^2^, cube=t^3^, sin1=sin[(2πt)/(365.25/7)], cos1=cos[(2πt)/(365.25/7)], sin2=sin[(4πt)/(365.25/7)], cos2=cos[(4πt)/(365.25/7)], H1=A(H1N1)_t_, H3=A(H3N2)_t_, H1pdm=A(H1N1)pdm_t_, B= B_t._.

Information on model selection for each outcome:

All-cause Deaths:

- For all cause deaths for the >=65 year age group, we selected the model including time, time-squared, sine, cosine, and the four influenza virus terms to predict influenza-associated excess deaths although the AIC value was lower in the model including the sine2 and cosine2 variables. We selected this model because only one of the additional variables had a statisticially significant association with the outcome, wheras in the model with only sine and cosine, the variables are either associated or very close to being associated with the outcome.

- For all-cause deaths for the <65 year age group, we selected the same model as the >= 65 year age group because the additional terms did not contribute significantly to the model. Both of these additional variables were not statistically significant. While the AIC using these variables was lower, they did not impact the beta values for the other variables in the model and thus did not likely impact the final outcomes and did not contribute to better estimation of influenza-associated deaths.

Respiratory and Circulatory Deaths:

- For R&C deaths for the <65 year age group, we selected the model including time, time-squared, sine, cosine, and the four influenza virus terms to predict influenza-associated excess deaths. This model had the lowest AIC value although it only differed slightly from other models examined. Further, the addition of the sine2 and cosine2 variables were not statistically significantly associated with the outcome of interest and did not impact the estimation of the beta values and thus did not appear to contribute to significantly to the estimation of influenza-associated excess deaths.

- For R&C deaths for the >=65 year age group, we selected the same model although the AIC value was lower in the model including the sine2 and cosine2 variables. We selected this model because only one of the additional variables had a statisticially significant association with the outcome, wheras in the model with only sine and cosine, the variables are either associated or very close to being associated with the outcome. Additionally, these extra harmonic variables did not appear to contribute to the estimation of betas and thus would not likely impact our estimation of influenza-associated mortality.
